# NET balancing: a problem in inflammatory lung diseases

**DOI:** 10.3389/fimmu.2013.00001

**Published:** 2013-01-24

**Authors:** Olivia Z. Cheng, Nades Palaniyar

**Affiliations:** ^1^Program in Physiology and Experimental Medicine, Lung Innate Immunity Research Laboratory, SickKids Research InstituteToronto, ON, Canada; ^2^Department of Laboratory Medicine and Pathobiology, University of TorontoToronto, ON, Canada; ^3^Institute of Medical Sciences, University of TorontoToronto, ON, Canada

**Keywords:** neutrophil extracellular traps (NETs), lung inflammation, lung infection, surfactant protein D (SP-D), cystic fibrosis (CF), acute lung injury (ALI), neutrophil

## Abstract

Neutrophil extracellular traps (NETs) are beneficial antimicrobial defense structures that can help fight against invading pathogens in the host. However, recent studies reveal that NETs exert adverse effects in a number of diseases including those of the lung. Many inflammatory lung diseases are characterized with a massive influx of neutrophils into the airways. Neutrophils contribute to the pathology of these diseases. To date, NETs have been identified in the lungs of cystic fibrosis (CF), acute lung injury (ALI), allergic asthma, and lungs infected with bacteria, virus, or fungi. These microbes and several host factors can stimulate NET formation, or NETosis. Different forms of NETosis have been identified and are dependent on varying types of stimuli. All of these pathways however appear to result in the formation of NETs that contain DNA, modified extracellular histones, proteases, and cytotoxic enzymes. Some of the NET components are immunogenic and damaging to host tissue. Innate immune collectins, such as pulmonary surfactant protein D (SP-D), bind NETs, and enhance the clearance of dying cells and DNA by alveolar macrophages. In many inflammatory lung diseases, bronchoalveolar SP-D levels are altered and its deficiency results in the accumulation of DNA in the lungs. Some of the other therapeutic molecules under consideration for treating NET-related diseases include DNases, antiproteases, myeloperoxidase (MPO) inhibitors, peptidylarginine deiminase-4 inhibitors, and anti-histone antibodies. NETs could provide important biological advantage for the host to fight against certain microbial infections. However, too much of a good thing can be a bad thing. Maintaining the right balance of NET formation and reducing the amount of NETs that accumulate in tissues are essential for harnessing the power of NETs with minimal damage to the hosts.

## Introduction

Although neutrophils are critical to our immune system in the event of microbial infections, an overabundance of neutrophils in circulation or in tissues has been implicated to be a problem in a number of lung diseases. Patients with inflammatory lung diseases such as cystic fibrosis (CF), severe asthma, chronic obstructive pulmonary disease (COPD), acute lung injury (ALI), acute respiratory distress syndrome (ARDS), and emphysema all exhibit various degrees of neutrophil influx; these neutrophils are a major contributor to these diseases (Downey et al., 2009; Grommes and Soehnlein, 2011). A massive influx of neutrophils is seen in acute pulmonary infections, pneumonia, and sepsis. Many of these lung conditions lead to ALI and tissue damage (Grommes and Soehnlein, 2011). Neutrophils and neutrophil extracellular traps (NETs) found in these inflammatory conditions cause tissue injury and severe inflammation in the lung (Villanueva et al., 2011; Saffarzadeh et al., 2012). NETs are extracellular DNA complexed with antimicrobial proteins, and help to fight infectious agents. However, an excess of NETs contributes to the pathology of a number of diseases. In the lungs, NETs have been identified in conditions of CF (Manzenreiter et al., 2012), ALI (Thomas et al., 2012), and infections with bacteria (Douda et al., 2011b), fungi (Bruns et al., 2010), and viruses (Narasaraju et al., 2011; Ng et al., 2012). In this review, neutrophil and NET functions during inflammation and infection will be discussed, followed by their contribution to tissue injury, autoimmunity, ALI, CF, and asthma. Lastly, we will discuss the targeting of NETs in therapy.

## Neutrophil function and recruitment during inflammation and infection

Neutrophils are an important component of our host defense against invading pathogens, often referred to as the immune system's first line of defense against infection. The neutrophil is the most abundant leukocyte comprising approximately 60% of all leukocytes found in circulating blood in humans. They are easily identified by their banded or multi-lobed nuclear structure, thus giving them their synonymous name of polymorphonuclear leukocytes (PMNs) (Nathan, 2006). Many of these neutrophils enter the lungs during infections and form NETs. Dysfunctions in NETosis and NET clearance can severely damage this vital organ.

### Antimicrobial mechanisms of neutrophils

The general dogma was that neutrophils fight against microorganisms by directly phagocytosing the targets or by releasing toxic components via degranulation. Phagocytosis is one of the mechanisms identified in neutrophils that can directly engulf and digest potential pathogens as well as cell debris. Internalized pathogens are contained in phagosomes, where antimicrobial peptides from cellular granules and reactive oxygen species (ROS) produced by NADPH oxidase work together to create a toxic environment for most pathogens (Underhill and Ozinsky, 2002). Degranulation is the release of toxic ROS and antimicrobial granular proteins into the extracellular space. Neutrophil granules are categorized into three different types based on their contents: primary (azurophilic), secondary (specific), and tertiary (gelatinase). The presence of different types of granules in the neutrophils is dependent on the time of granule formation relative to the neutrophil maturation stage. This starts with the formation of primary granules, followed by secondary and tertiary granules (Gullberg et al., 1997). Primary granules contain MPO, elastase, cathepsin G, proteinase 3, defensins, and lysozyme; secondary granules contain collagenase, gelatinase, cystatin, lysozyme, and lactoferrin; tertiary granules contain gelatinase, lysozyme, and arginase. As such, a neutrophil will accumulate all three types of granules by the end of maturation. Collectively, these granules contain many antimicrobial proteins that function to fight infection in the lungs and other organs (Borregaard et al., 2007).

Neutrophils also indirectly defend the host against microbes by participating in elaborate cell signaling networks involving cytokines, chemokines, survival and growth factors that cause downstream pro-inflammatory effects. Neutrophils can secrete pro-inflammatory cytokines (e.g., TNF-α, IL-1β), CC and CXC chemokines (e.g., IL-8, IFN, IP-10, MIP-1α). The secretion itself is regulated by immunoregulatory cytokines (e.g., IFN-γ, IL-4, IL-13, IL-10) (Kasama et al., 2005). These factors can increase the production of various chemokines and cytokines to further regulate neutrophil functions (Kato and Kitagawa, 2006). Importantly, some of these factors can participate in recruiting more neutrophils or other leukocytes to the site of infection or sterile inflammation (Cassatella et al., 1997).

### Neutrophil migration into the lungs

Typically, neutrophils are found in higher concentrations in the pulmonary capillaries compared to systemic blood even in the absence of inflammatory stimuli. This phenomenon allows neutrophils to readily migrate into the lungs in response to inflammatory stimuli. Neutrophils undergo cellular deformation in order to emigrate between endothelial cells of the pulmonary capillaries to reach the alveolar air space (Doerschuk et al., 1999). During inflammation, neutrophils become activated upon stimulation and may undergo processes of ROS production, degranulation, NETs formation, or other functions. Activation of neutrophils is required before migration into the lungs (Ley et al., 2007). Neutrophil activating factors may be derived from host [e.g., platelet activating factor (PAF), leukotriene B4, IL-8] or from pathogens [e.g., formylated peptide (fMLP) and lipopolysaccharide (LPS) (Krause et al., 1985; Martin et al., 1989; Anderson et al., 1991; Corteling et al., 2002; Mukaida, 2003)]. The chemokines that are most critical for neutrophil recruitment in the lungs include IL-8 (CXCL8) in humans, and MIP-2 (CXCL2) and KC (CXCL1) in rodents (Kobayashi, 2008). These chemokines are secreted by neutrophils themselves, epithelial cells, or macrophages (Cassatella et al., 1997; Matsukawa and Yoshinaga, 1999; Yamashiro et al., 2001; Kasama et al., 2005; Kato and Kitagawa, 2006).

## NETs

**Figure 1:**
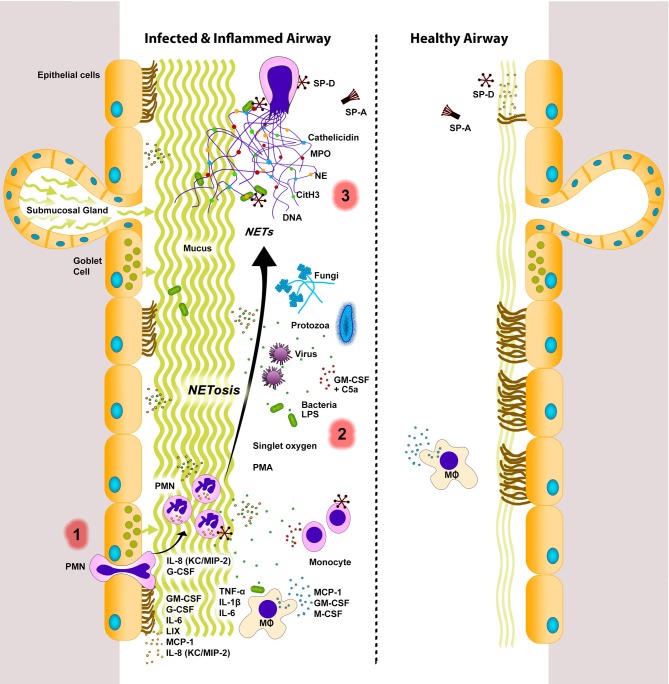
**NETs in infected and inflamed airways.** Lungs respond to sterile injury or infection by secreting various signaling molecules. During infection and inflammation, various cells (e.g., endothelial, epithelial, immune cells) express inflammatory cytokines, chemokines, and growth factors to recruit monocytes (e.g., MCP-1) and neutrophils [e.g., IL-8 (KC/MIP-2)] into the airway lumen. Neutrophils can be stimulated by a variety of agents (e.g., bacteria, viruses, fungi, protozoa, LPS, singlet oxygen, PMA, GM-CSF+C5a) to undergo NETosis. Release of cytotoxic DNA–protein complexes [e.g., citrullinated histone (CitH_3_), neutrophil elastase (NE), myeloperoxidase (MPO), cathelicidin, other neutrophil proteases] not only increase mucus viscosity, but also contribute to lung damage that can perpetuate the vicious cycle of lung injury and inflammation. NETs are considered to be degraded by DNase enzymes. Macrophages can also internalize and remove DNA, as well as other cellular debris. A balance between NETosis and NET clearance is essential for effectively clearing infectious agents with minimal damage to the lungs. Dysregulation in these two processes can lead to lung injury and exacerbation of lung diseases. Innate immune collectins could help to maintain healthy lungs with minimal inflammation. SP-A, pulmonary surfactant protein A; SP-D, pulmonary surfactant protein D. PMN, neutrophils; MΦ, macrophages. Inflamed airways also have excess mucus. The putative sequence of NETotic events in the lungs are numbered as 1, 2, and 3. Note: Cytokines, chemokines, and growth factors are placed near their most probable source of secretion. However, the source and/or degree of cytokine secretion varies depending on the stimuli.

Aside from the more traditional mechanisms of phagocytosis and degranulation, neutrophils can also generate NETs to directly combat microbes during inflammation and infection (Brinkmann et al., 2004). Takei et al. first described this novel form of neutrophil cell death to be distinct from apoptosis and necrosis in 1996 (Takei et al., 1996). This was later studied by Brinkmann et al. (2004), who coined the term NETosis for this cell death process. NETs are cast as decondensed chromatin fibers coated with antimicrobial histones and granular proteins (Brinkmann et al., 2004) (Figure 1). To date, NETs and NET-like structures have been identified by several labs as a host defense mechanism in many organisms including humans (Manzenreiter et al., 2012), mice (Ermert et al., 2009a), chickens (HETs) (Chuammitri et al., 2009), cats (Wardini et al., 2010), cattle (Aulik et al., 2010), fish (Palić et al., 2007b), insects (Altincicek et al., 2008), and even plants (Wen et al., 2009). Conservation of NET function across species suggests an evolutionary advantage of NETs in immune defense.

### NET induction

The formation of NETs (NETosis) is stimulated by a variety of agents (Table 1). Microorganisms such as protozoa (Guimarães-Costa et al., 2009; Abi Abdallah et al., 2012), fungi (Urban et al., 2006, 2009; Ermert et al., 2009b), viruses (Narasaraju et al., 2011; Ng et al., 2012; Saitoh et al., 2012), bacteria (Brinkmann et al., 2004; Crotty Alexander et al., 2010), and bacterial component LPS (Douda et al., 2011b) can induce NETosis. Host-derived factors such as granulocyte/macrophage colony-stimulating factor (GM-CSF) with complement factor 5a (Yousefi et al., 2009), activated platelets (Clark et al., 2007; Caudrillier et al., 2012) and singlet oxygen (Nishinaka et al., 2011) also induce NETosis (Table 1). The pharmacological agent phorbol-12-myristate-13-acetate (PMA), a protein kinase C activator, is a known strong inducer of NETosis that is routinely used in studies of NETs. The potent neutrophil chemoattractant, IL-8, has also been shown to induce NETosis (Brinkmann et al., 2004; Gupta et al., 2005), but there has been some uncertainty regarding its ability to trigger NETosis in CF airways (Marcos et al., 2010, 2011).

**Table 1:** **NETosis-inducing agents**.

**NETosis inducer**	***In vitro***	***In vivo***	**Reference(s)**
**BACTERIA**			
*Escherichia coli (P4 strain)*	10 MOI; bovine	–	Clark et al., 2007; Grinberg et al. 2008; Yost et al., 2009
	0.01 MOI; human	–	
*Pseudomonas aeruginosa (PA01)*	0.1–10 MOI; human	1 × 10^6^ CFU/mouse	Douda et al., 2011b; Young et al., 2011
*Staphylococcus aureus*	0.01–10 MOI; human	–	Brinkmann et al., 2004; Pilsczek et al., 2010
*Shigella flexneri*	0.01 MOI; human	2.5–3.0 × 10^10^/rabbit	Brinkmann et al., 2004
*Salmonella enteric*	0.01 MOI; human	–	Brinkmann et al., 2004
Group A *Streptococcus*	0.1 MOI; human	5 × 10^7^–2 × 10^8^ CFU/mouse	Buchanan et al., 2006; Crotty Alexander et al., 2010
*Streptococcus pneumonia*	0.01 MOI; human	1 × 10^7^/mouse	Beiter et al., 2006
*Mycobacterium tuberculosis*	0.1–10 MOI; human	–	Ramos-Kichik et al., 2009
**PROTOZOA**			
*Leishmania amazonensis*	10 MOI; human	–	Guimarães-Costa et al., 2009
*Leishmania donovani*	10 MOI; human	–	Gabriel et al., 2010
*Toxoplasma gondii*	250 mU/ml; human, mouse	5 × 10^7^/mouse	Abi Abdallah et al., 2012
*Eimeria bovis*	0.2 (sporozoites) MOI; bovine	–	Behrendt et al., 2010
**FUNGI**			
*Aspergillus fumigates*	5 (conidia) MOI; human	–	Bruns et al., 2010
*Candida albicans*	0.01 MOI; human	–	Urban et al., 2006
*Aspergillus nidulans*	0.5 (conidia) MOI; human	–	Bianchi et al., 2009
**VIRUS**			
Human immunodeficiency virus (p24 antigen)	1.0–2.4 ng/ml; human	–	Saitoh et al., 2012
Influenza A virus H1N1	20 MOI; human	100–500 PFU/mouse	Narasaraju et al., 2011
Influenza A virus H3N2	2 MOI; mouse	2 × 10^5^ PFU/mouse	Ng et al., 2012
**HOST FACTORS**			
GM-CSF + C5a	25 ng/ml GM-CSF + 10^−7^ M C5a	–	Yousefi et al., 2009
IL-8 (CXCL8)	2.5–10 ng/ml; human	–	Gupta et al., 2005
MIP-2 (CXCL2)	100 nM; human	–	Marcos et al., 2010, 2011
Singlet oxygen	10 μg/ml Photofrin; human	–	Nishinaka et al., 2011
Platelet activating factor (PAF)	10^−10^ − 10^−7^ M; human	–	Yost et al., 2009
Syncytiotrophoblast microparticles (STBM)	150 μg/ml; human	–	Gupta et al., 2005
**OTHERS**			
Glucose oxidase	200–1000 mU/ml; human	–	Yost et al., 2009
Calcium ionophore (ionomycin)	5 μg/ml; zebrafish 4 μM; human	–	Palić et al., 2007a; Neeli et al., 2008
Phorbol-12-myristate-13-acetate (PMA)	25–100 nM; human	–	Brinkmann et al., 2004; Remijsen et al., 2011
Bacterial component LPS, Panton-Valentine leukocidin	100 ng/ml; human	5–25 μg/mouse	Brinkmann et al., 2004; Clark et al., 2007; Pilsczek et al., 2010; Douda et al., 2011b

### NETosis mechanism

The process of NETosis requires mature neutrophils (Martinelli et al., 2004) and the presence of enzymes MPO, neutrophil elastase (NE), and peptidylarginine deiminase type IV (PAD4) (Neeli et al., 2008; Wang et al., 2009; Papayannopoulos et al., 2010; Metzler et al., 2011). Upon stimulation of the neutrophil, the nuclear envelope disintegrates to allow mixing of chromatin with granular proteins (Brinkmann et al., 2004; Fuchs et al., 2007). NE and MPO degrade histones and promote chromatin decondensation (Papayannopoulos et al., 2010). PAD4 mediates chromatin decondensation by hypercitrullinating positively charged arginines of specific histones to relieve electrostatic coiling of the chromatin (Wang et al., 2009; Li et al., 2010; Leshner et al., 2012). These DNA–protein complexes are then released extracellularly as NETs.

As the discovery of NETs is relatively new, the mechanism of NETosis is not clearly understood. The majority of studies reveal that NETosis is dependent on the generation of ROS by NADPH oxidase; however, a few studies show that NETosis may also occur in a ROS-independent manner, for instance by stimulation with *Staphylococcus aureus* (Pilsczek et al., 2010). Patients with chronic granulomatous disease (CGD) have congenital defects in different subunits of NADPH oxidase (Nox2) that prevent their ability to generate ROS. Hence, the neutrophils of these patients are unable to perform phagocytic killing and NETosis, making them highly susceptible to life-threatening infections (Fuchs et al., 2007). The restoration of NADPH oxidase function and NET formation in these patients effectively protected them against microbial infections (Bianchi et al., 2009). Singlet oxygen is a member of the ROS family that has been shown to be essential for the formation of NETs. Singlet oxygen itself can trigger NETosis independent of NADPH oxidase (Nishinaka et al., 2011). In addition to superoxide, autophagy has also been shown to be required for the generation of NETs (Remijsen et al., 2011). Recent evidence shows that the NETosis pathway requires cell signaling, of which p38 MAP kinase and Raf-MEK-ERK kinase pathways are involved (Hakkim et al., 2011; Keshari et al., 2012). Nonetheless, depending on the stimulus, the key components involved in the generation of NETs can vary (Parker et al., 2012) (Table 2).

**Table 2:** **Neutrophil components involved in NETosis**.

**Component**	**Function**	**Reference(s)**
Neutrophil elastase (NE)	Chromatin decondensation	Papayannopoulos et al., 2010
Myeloperoxidase (MPO) HOCl	Chromatin decondensation; hypochlorite generation	Papayannopoulos et al., 2010; Metzler et al., 2011; Akong-Moore et al., 2012
Peptidylarginine deiminase type IV (PAD4)	Chromatin decondensation; histone modification	Li et al., 2010; Leshner et al., 2012
Autophagy	NETosis pathway	Remijsen et al., 2011
NADPH oxidase	NETosis pathway	Fuchs et al., 2007
H_2_O_2_	Substrate for MPO	Akong-Moore et al., 2012
Singlet oxygen	Essential NETosis inducer	Nishinaka et al., 2011
Raf-MEK-ERK	NETosis pathway	Hakkim et al., 2011
ERK, p38 MAPK	NETosis pathway	Keshari et al., 2012

### Alternative types of extracellular traps

Extracellular DNA traps have been more recently documented to be not exclusive to only neutrophils. Extracellular DNA traps can also be generated from macrophages (METs) (Hellenbrand et al., 2013), eosinophils (EETs) (Yousefi et al., 2008; Dworski et al., 2011), and mast cells (MCETs) (von Köckritz-Blickwede et al., 2008; Lin et al., 2011). Nonetheless, all extracellular DNA traps are of an immune cell origin that contains a plethora of antimicrobial components. Most consider NETosis as a form of cell death distinct from classical apoptosis and necrosis since it requires histone hypercitrullination; however, the term “cell death” may not be entirely appropriate. In the late 1980's, Malawista et al. of Yale showed that enucleated neutrophils (i.e., cytoplasts) remain viable and are capable of killing microbes (Malawista et al., 1989). Recent studies also corroborate that cells do not necessarily die after the release of extracellular DNA traps (Yousefi et al., 2009; Pilsczek et al., 2010). Yipp et al. recently showed that neutrophils that undergo NETosis without lysis are viable and retain their ability to phagocytose bacteria (Yipp et al., 2012). In another study, neutrophils were viable after being primed with GM-CSF, then stimulated with LPS or C5a to release NETs of mitochondrial origin (Yousefi et al., 2009). A similar study by the same group showed that eosinophils were also viable after the release of EETs of mitochondrial origin (Yousefi et al., 2008). The reasoning for the viability of these cells after the release of extracellular DNA traps is thought to be caused by the type of DNA released; only mitochondrial DNA was extruded while nuclear DNA remained intact within the nucleus of the cell to allow neutrophils to continue its function. However, recent studies challenged this idea (Pilsczek et al., 2010; Yipp et al., 2012). In these studies, neutrophils remained viable after the release of NETs that are of nuclear origin. Once these neutrophils were stimulated with *S. aureus*, the neutrophils underwent a novel mechanism of rapid NETosis. NETs were released via a vesicular mechanism, in which vesicles budding from the neutrophil contained nuclear DNA (Pilsczek et al., 2010). However, the stimuli used for these studies were different. Depending on the stimulus, neutrophils can undergo a different form of NETosis (Parker et al., 2012).

## NET-mediated tissue injury and diseases

Despite the advantageous properties of NETs, their ineffective clearance and regulation can have pathological effects (Figure 1). The antimicrobial histones and peptides coating the NET-DNA are directly cytotoxic to tissue, and ineffective clearance of NETs causes deleterious inflammation of host tissue. NETs, and in particular extracellular histones, can directly cause epithelial and endothelial cell death (Xu et al., 2009; Saffarzadeh et al., 2012). Histone administration *in vivo* resulted in neutrophil margination, vacuolated endothelium, intra-alveolar hemorrhage, and macro- and microvascular thrombosis (Xu et al., 2009). Impaired degradation and clearance of NETs has also been shown to be linked to autoimmunity in patients with atherosclerosis (Döring et al., 2012), rheumatoid arthritis (Rohrbach et al., 2012), small-vessel vasculitis (SVV) (Kessenbrock et al., 2009), systemic lupus erythematosus (SLE) (Hakkim et al., 2010; Lande et al., 2011; Leffler et al., 2012; Liu et al., 2012), and Felty's syndrome (Dwivedi et al., 2012). PAD4 citrullinated histones in particular are highly immunogenic (Neeli et al., 2008). Autoantibodies against these modified histones are seen in patients with SLE (Liu et al., 2012), Felty's syndrome (Dwivedi et al., 2012) and a mouse model of rheumatoid arthritis (Rohrbach et al., 2012). The presence of autoantibodies in chronic inflammatory lung diseases has not been investigated, but the prolonged presence of NETs in the lungs may potentially elicit autoimmune responses.

In SLE patients, the self-DNA and antimicrobial peptides of NETs are immunogenic complexes that can activate plasmacytoid dendritic cells (pDCs) and serve as autoantigens to B cells in their production of anti-NET autoantibodies (Lande et al., 2011). Both anti-NET antibodies and DNase 1 inhibitors were found in the sera of SLE patients; these inhibitors prevented DNase 1 to access NETs for degradation (Hakkim et al., 2010). C1q deposited on NETs have also been shown to prevent NET degradation by directly inhibiting DNase 1 (Leffler et al., 2012). The deposition of C1q on NETs can activate complement to cause further neutrophil recruitment (Stokol et al., 2004; Leffler et al., 2012), which can further exacerbate the disease. Similarly in atherosclerosis, self-DNA and antimicrobial peptides of NET structures are auto-antigenic and stimulate pDC-driven autoimmunity via TLR7/9 and production of type I IFN (Döring et al., 2012). As NETs derive autoantibodies, they can also form soluble immune complexes (ICs), which is hallmark of autoimmune diseases. Recently, Chen et al. showed that ICs can induce NETosis in mice *in vivo* via FcγRIIA independent of NE, MPO, and NADPH oxidase (Chen et al., 2012). This study implicates that FcγR may play an important role in the NETosis pathway.

In SVV, anti-neutrophil cytoplasmic autoantibodies (ANCAs) are strongly associated with the disease (Kallenberg et al., 2006). Similar to ICs in SLE patients, ANCAs directed against proteinase-3 (PR3) and MPO can stimulate neutrophils in SVV to form NETs and promote autoimmunity (Sangaletti et al., 2012). These neutrophil proteins (PR3, MPO) are found attached to the chromatin scaffold of NETs and may be the host antigen source for the generation of ANCAs. The enhanced deposition of antimicrobial peptide LL37 (cathelicidin) onto NET-DNA was also observed in SVV (Kessenbrock et al., 2009). The binding of LL37 to NET-DNA can protect it from degradation (Lande et al., 2011) and has been shown to drive the autoimmune response and pathogenesis of SLE and psoriasis (Lande et al., 2007). As such, LL37 may have a role in the autoimmunity and pathogenesis of SVV as well as other NET-related diseases. All in all, these highly immunogenic NET structures result in the production of autoantibodies, further neutrophil recruitment and triggering of NETosis, which create a perpetuating cycle of autoimmune combat. Clearance of NETs from the lungs and other sites are essential for preventing NET-associated tissue and organ damage.

## NETs and surfactant protein D (SP-D)

The lungs are lined with a pulmonary surfactant layer that contains surfactant proteins (SP-) A and D. These proteins help to prevent the lungs from infection and inflammation, especially because airways are constantly exposed to microorganisms and debris. SP-A and SP-D are innate immune collectins that can opsonize pathogens, and apoptotic and necrotic cells to signal their clearance by alveolar macrophages in the lungs and modulate pulmonary inflammation (Nayak et al., 2012). Specifically, SP-A and SP-D contain carbohydrate recognition domains and collagenous domains that can bind carbohydrate ligands of bacteria and DNA, respectively (Palaniyar et al., 2003a, 2004; Litvack and Palaniyar, 2010). The binding of these surfactant proteins to DNA and apoptotic cells enhances their clearance by alveolar macrophages (Schagat et al., 2001; Palaniyar et al., 2003a,b, 2005). SP-D in particular has a role in reducing apoptosis of alveolar macrophages and pro-inflammatory cytokines (Clark et al., 2002, 2003). As such, SP-A and SP-D have important roles in maintaining infection- and inflammation-free airways.

Recently, our lab showed that SP-D could simultaneously bind both NET-DNA and bacteria to help microagglutinate bacteria and promote bacterial trapping by NETs (Douda et al., 2011b). Currently, the factors that can suppress NETosis and promote the clearance of NETs are unknown. The binding of SP-D to DNA enhances the clearance of DNA by macrophages (Palaniyar et al., 2005); however, the role of SP-D on NET-DNA clearance is not clear. Preliminary studies from our lab suggest that SP-D can augment the clearance of NETs by alveolar macrophages (Douda et al., 2011a). There are a number of human inflammatory lung diseases that are characterized by decreased levels of bronchoalveolar SP-D. SP-D deficiency can lead to the accumulation of dying cells and increased production of anti-DNA auto-antibodies (Palaniyar et al., 2005). These studies suggest that SP-D is one of the important proteins for maintaining a balance of NETs in the lungs.

## Acute lung injury (ALI) and acute respiratory distress syndrome (ARDS)

Infection-related conditions such as pneumonia, sepsis, and pulmonary infections with viruses, bacteria, or fungi can directly injure the lungs and cause ALI or ARDS. Non-infectious causes (sterile injury) such as high-tidal ventilation, hyperoxia, and pulmonary contusions also lead to ALI and ARDS (Matthay et al., 2012). ALI is described as a lung disease with acute onset and disruption of the alveolar-capillary interface that leads to increased microvascular permeability. As a result, protein-rich fluid from the capillaries leaks into the alveolar space causing pulmonary edema. ALI and ARDS have many different causes, but epithelial injury is the basis of ARDS, and it is a more severe form of ALI (Zhou et al., 2012). ALI/ARDS is characterized by a massive influx of neutrophils into the lungs causing neutrophilic inflammation. Excessive activation and migration of neutrophils into the lung is a hallmark of ALI. Neutrophils are important contributors to the progression of ALI/ARDS, and higher neutrophil concentration in the BAL fluid of patients with ARDS is often associated with greater severity of the disease (Grommes and Soehnlein, 2011). Excessive neutrophils and NETs contribute to the pathology of ALI, where NETs can directly induce lung epithelial cell death (Saffarzadeh et al., 2012).

NETs are also found in infection-related ALI models of influenza virus (Narasaraju et al., 2011; Ng et al., 2012), bacteria or bacterial component LPS (Li et al., 2010; Douda et al., 2011b; Barletta et al., 2012), and fungi (Urban et al., 2006, 2009; Hosogi et al., 2008; Bruns et al., 2010). Toll-like receptor 4 (TLR4) is a well-characterized pathogen recognition receptor that recognizes pathogen-associated molecular patterns found on pathogens such as viruses, fungi, and bacteria to initiate an immune response (Noreen et al., 2012). LPS is an important ligand of TLR4 that has been routinely shown to cause NETosis (Douda et al., 2011a; Barletta et al., 2012). In the presence of LPS, activated platelets containing TLR4, but not TLR4-deficient platelets migrate into the lungs (Andonegui et al., 2005). These activated platelets can bind to neutrophils to elicit neutrophil activation and induce NETosis (Clark et al., 2007; Caudrillier et al., 2012).

NETs can also be found in ALI models of sterile injury such as transfusion-related ALI (TRALI) (Caudrillier et al., 2012; Thomas et al., 2012). Plasma NETs are found in both ALI and TRALI patients. In addition to NETs, TRALI patients also have the antibody against human neutrophil alloantigen-3a (HNA-3a) in their blood. HNA-3a causes the most severe TRALI and has been shown to promote NETosis in human neutrophils *in vitro* (Thomas et al., 2012). Activated platelets have been shown to induce NETosis not only in TRALI, but also in severe sepsis and deep vein thrombosis (Clark et al., 2007; Brill et al., 2012; Caudrillier et al., 2012; Fuchs et al., 2012). NETs provide a platform for platelets to promote coagulation, thrombosis, and inflammation in vascular diseases such as atherosclerosis, sepsis, and thrombotic diseases (e.g., cancer-associated thrombosis) (Demers et al., 2012). As activated platelets can trigger NETosis, the histone/DNA complexes of NETs too can activate platelets that further promote NETosis, thrombosis, and coagulation (Semeraro et al., 2011).

Neutrophils and platelets are both key players to the ALI pathology. In a TRALI mouse model, depletion of either neutrophils or platelets was protective (Looney et al., 2009). Comparably, the use of either aspirin or a glycoprotein IIb/IIIa inhibitor to target platelet activation effectively decreased NET formation and lung injury. To target NETs, a histone-blocking antibody and DNase 1 were used and shown to be protective against TRALI (Caudrillier et al., 2012). DNase 1 treatment alone during TRALI was able to improve blood oxygenation and prevent alveolar accumulation of NETs (Thomas et al., 2012). As such, targeting NETs may be a promising therapeutic approach in the treatment of ALI.

The extracellular DNA found accumulated in the airways of LPS-induced ALI and TRALI mice *in vivo* are attributed to NETs (Douda et al., 2011b; Caudrillier et al., 2012; Thomas et al., 2012). SP-D levels in BALF are reduced in patients with ARDS, children with respiratory syncytial virus (RSV) infection, and LPS-induced ALI mouse models (Hartl and Griese, 2006; Douda et al., 2011b). Decreased levels of SP-D may play a contributing factor to the impaired clearance of DNA from these lungs (Palaniyar et al., 2005; Douda et al., 2011b).

## Cystic fibrosis (CF)

Another lung disease featuring chronic airway infections is CF. CF is caused by mutations in the CF transmembrane conductance regulator (CFTR) gene (Riordan et al., 1989), of which CF lung disease is the major cause of morbidity and mortality in these patients (Ratjen and Grasemann, 2012). CFTR is responsible for the modulation of bicarbonate and chloride secretion across airway epithelial cells, as well as for the regulation of sodium absorption via epithelial sodium channel (ENaC) (Stutts et al., 1997; Coakley et al., 2003; Berdiev et al., 2009). Patients with CF have impaired ion transport across the epithelium that ultimately leads to dehydration of the airway surface liquid (Matsui et al., 1998). Consequently, there is increased mucus viscosity and impaired mucociliary clearance (Henke and Ratjen, 2007). There are vast amounts of free DNA that accumulate in CF lungs that contribute to the increased mucus viscosity found in their airways (Henke and Ratjen, 2007). The DNA levels in their airways correlates with neutrophil count, and can be used as an index to inflammation and lung disease severity (Kirchner et al., 1996; Ratjen et al., 2005). Severe neutrophilic inflammation and dying of neutrophils is characteristic of CF lung disease. The origin of the DNA found in CF airways has been traditionally considered to be from necrotic neutrophils (Lethem et al., 1990). Studies conducted after the discovery of NETs have challenged this idea suggesting that the DNA is attributed to NETs as opposed to necrotic neutrophils (Marcos et al., 2010, 2011; Manzenreiter et al., 2012). Understanding the mechanisms that regulate neutrophil death in these airways will facilitate the identification of new therapeutic targets.

Although neutrophils and NETs play vital beneficial roles against infection, their success in host defense in CF patients is significantly compromised as patients often suffer chronic bacterial infections in their lungs. The microbiota present in CF airways is diverse, but eventual chronic pulmonary infections are dominated by opportunistic pathogens *Pseudomonas aeruginosa* and *Burkholderia cepacia* (Razvi et al., 2009; Fodor et al., 2012). In addition to the inability of neutrophils and NETs to eradicate bacteria, the DNA released from neutrophils can promote bacterial colonization and biofilm formation (Parks et al., 2009; Fuxman Bass et al., 2010). CF neutrophils can cast NETs against *P. aeruginosa*. However, evidence reveals that clinical strains of *P. aeruginosa* can acquire resistance to NET-mediated killing over the course of infection in CF airways (Young et al., 2011). The accumulation of bacteria, extracellular DNA and NET-associated enzymes such as MPO and elastases (neutrophil elastase, *Pseudomonas* elastase) worsen lung inflammation and tissue damage (Elizur et al., 2008; Voynow et al., 2008; Gupta et al., 2010; Xu et al., 2011; Dubois et al., 2012; Saffarzadeh et al., 2012). NE in the lungs can further exacerbate inflammation by inducing IL-8 expression for the recruitment of even more neutrophils (Nakamura et al., 1992). Neutrophils in CF airways exhibit a dysfunctional phenotype (Tirouvanziam et al., 2008). The gene expression profile and activation states of CF neutrophils and wild-type neutrophils are different (Adib-Conquy et al., 2008; Tirouvanziam et al., 2008; McKeon et al., 2010; Su et al., 2011), but the implications of these differences on neutrophil or NET function are not clearly understood.

Why more NETosis occurs in CF airways is unknown. Early stage CF lung disease is predominated by inflammation in the absence of any detectable infectious agents. At this stage, NETs are likely induced by host factors. As the lungs of CF patients are chronically infected with bacteria at later stages, it is likely that the source of NETosis stimulation may also be derived from bacterial components. The common pathogens (e.g., *S. aureus, P. aeruginosa, A. fumigatus, C. albicans*) that colonize the lungs of CF patients have been shown to be effective inducers of NETosis (Urban et al., 2006; Bruns et al., 2010; Pilsczek et al., 2010; Young et al., 2011). However, there is still debate on whether inflammation is secondary to chronic infection or vice versa (Becker et al., 2004; Verhaeghe et al., 2007). A number of studies reveal that inflammation and accumulation of neutrophils is seen early on in CF airways prior to the presence of any apparent infection (Tirouvanziam et al., 2000; Verhaeghe et al., 2007). Early CF airways have increased NF-κB activation and inflammatory cytokines such as IL-8, TNF, and GM-CSF (Khan et al., 1995; Rosenfeld et al., 2001; Verhaeghe et al., 2007). NF-κB is an inducible transcription factor that plays a key role in the regulation of cytokines and chemokines, cell adhesion molecules, acute phase proteins, and anti-microbial peptides during pulmonary inflammation (Batra et al., 2011). The contribution of these host-derived molecules on NETosis is unknown. IL-8 has been previously shown to induce NETosis in other studies (Brinkmann et al., 2004; Gupta et al., 2005, 2010), but its ability to induce NETosis in CF airways is uncertain (Marcos et al., 2010, 2011).

SP-A and SP-D levels are decreased in CF patients, where their concentration is inversely related to the degree of inflammation in early CF disease (Postle et al., 1999; Noah et al., 2003). Additionally, there is an inverse relationship between SP-D level and neutrophil count in BALF (Griese et al., 2004). The lack of SP-D may have implications to the ineffective clearing of DNA in their lungs. The accumulation of NETs in the lung may lead to lung damage and exacerbate the disease by thickening the mucus layer. Aerosolized recombinant human DNase (rhDNase) is a therapeutic option used to treat patients with moderate to severe CF lung disease (Shak et al., 1990). It is used to break down polymerized DNA in the CF airways in order to reduce mucus viscosity. rhDNase treatment has shown to effectively reduce pulmonary exacerbations and improve lung function in some patients (Paul et al., 2004; Ratjen et al., 2005; Henke and Ratjen, 2007). However, DNase treatment does not help with the severe neutrophilic inflammation, chronic bacterial infection, and further deterioration of the lung. Neutrophils in CF lungs release uncontrolled extracellular proteases that destroy lung tissue, and exogenous protease inhibitors are ineffective in inhibiting these proteases (Griese et al., 2008; Voynow et al., 2008; Greene and McElvaney, 2009; Dubois et al., 2012). DNase can disrupt the ultrastructure of NETs, but DNase treatment can also dramatically increase the proteolytic activities of neutrophil enzymes (NE, cathepsin G, protease 3) bound to NETs (Dubois et al., 2012). Ultimately, NETs can harbor active proteases and protect these enzymes from exogenous protease inhibitors (Dubois et al., 2012). SP-D can be proteolytically degraded by active proteases HNE, *Pseudomonas* elastase, cathepsin G, and protease 3 *in vitro* (von Bredow et al., 2003). In CF lungs, SP-D is proteolytically damaged (Griese et al., 2003; von Bredow et al., 2003; Hirche et al., 2004), suggesting impaired host defense mechanisms of SP-D, which may contribute to the accumulation of NET-protein complexes and lung disease.

## Asthma

Asthma is a chronic disorder characterized by heterogeneous inflammation of the airways involving eosinophilic and non-eosinophilic phenotypes. Patients with neutrophilic asthma (i.e., greater proportion of neutrophils than eosinophils in sputum) usually have greater disease severity with reduced response to corticosteroid therapy (Simpson et al., 2006; Haldar and Pavord, 2007). A recent study showed that the only biomarkers that could distinguish severe or moderate asthma from mild asthma are neutrophil count and IL-8, out of the eight potential biomarkers (IL-8, neutrophils, eosinophils, IL-1Rα, IL-1α, IL-5, IL-6, and RANTES) investigated in BALF (Sur et al., 2012). IL-8 is a known chemoattractant for neutrophils. The neutrophilic inflammation observed in severe asthmatics may be attributable to the increased expression of IL-8 in airway smooth muscle cells, and the increased number of IL-8 positive cells found in epithelia (Pepe et al., 2005; Shannon et al., 2008).

Recently, extracellular DNA traps have been identified in allergic asthmatic airways (Dworski et al., 2011). In the atopic asthmatic airways, eosinophils predominated and were the source of extracellular DNA traps (EETs) observed. Similar to an earlier study on EETs (Yousefi et al., 2008), the DNA were of mitochondrial origin, not nuclear (Dworski et al., 2011). Subjects with neutrophilic asthma had higher neutrophil counts and NETs than eosinophils and EETs. IL-8, neutrophil count, and NETs are all increased in neutrophilic asthma, and their contribution to disease severity is not clearly understood. The cause of NETosis in asthmatic airways is unknown. IL-8 is a potential trigger of NETosis in these airways as it has been previously shown to induce NETosis in other studies (Brinkmann et al., 2004; Gupta et al., 2005, 2010). Plasma levels of activated platelets also increase during seasonal allergic rhinitis and asthma (Kasperska-Zajac et al., 2008). Since activated platelets are known inducers of NETosis, their elevated levels in plasma may imply a role in their contribution to NETs.

## Targeting NETs in therapy

The effective targeting of NET structures in therapy could benefit a multitude of diseases. The list of diseases associated with NETs has been constantly expanding since the discovery of NETs. This list includes SLE (Leffler et al., 2012), multiple sclerosis (Naegele et al., 2012), thrombotic diseases [cancer-associated thrombosis (Demers et al., 2012), deep vein thrombosis (Brill et al., 2012)], appendicitis (Brinkmann et al., 2004), sepsis (Clark et al., 2007), pre-eclampsia (Gupta et al., 2005), psoriasis (Lin et al., 2011), and HIV-1 (Saitoh et al., 2012). Current development of therapies to target NETs in inflammatory lung diseases include DNase (Shak et al., 1990; Hakkim et al., 2010), anti-histone antibodies (Xu et al., 2009, 2011; Semeraro et al., 2011), and antiproteases (Greene and McElvaney, 2009). DNase treatment is used for patients with ALI and CF to reduce pleural fluid viscosity by depolymerizing the DNA that accumulates in the lungs (Huggins et al., 2011). However, Dubois et al. showed that treating CF sputum with DNase could increase elastase activity (Dubois et al., 2012). Chronic inflammatory lung diseases already have elevated levels of proteases, which lead to lung damage and increased inflammation. Antiproteases are used in therapy to dampen the activity of these proteases (Greene and McElvaney, 2009). The use of exogenous protease inhibitors alone has been shown to be ineffective in CF sputum because NETs serve as a reservoir of these active proteases and protect them from inhibition (Dubois et al., 2012). As such, the combined use of DNase and antiproteases may be potentially helpful in controlling NET-mediated lung damage. The use of anti-histone antibodies has also been shown to be protective of NET-mediated lung damage in a TRALI mouse model (Caudrillier et al., 2012). This approach has yet to be investigated in humans; however, the use of anti-histone antibodies raises some concerns. Because extracellular histones are highly immunogenic and induce the production of autoantibodies (Liu et al., 2012), the use of anti-histone antibodies in therapy may promote autoimmunity. As an alternative, others have suggested using anionic polymers such as polysialic acid to neutralize histones (Saffarzadeh et al., 2012). MPO inhibitors have also been considered (Papayannopoulos et al., 2010). SP-D is another protein candidate that could regulate NETosis and NET clearance, and prevent autoantibody generations. Although an excess of NETs may lead to pathologies, moderate amounts are beneficial in protecting hosts against infections. Since NETosis involves components common to other essential pathways in the body (e.g., ERK pathway, p38 kinase pathway, autophagy pathway and intracellular microbial killing NADPH oxidases), careful consideration is required to design drugs to regulate NETosis.

## Conclusion

Neutrophil function and NETs are critical components of our immune defense. Patients with CGD have impaired neutrophil function and cannot form NETs, making them highly susceptible to lethal infections. Although NETs are important, an excess due to the dysregulation of NETosis can lead to many pathologies. Exaggerated neutrophil recruitment, activation, and NET formation are characteristic of inflammatory lung diseases like CF and ALI. The prolonged presence of NETs is extremely deleterious to host tissue and can stimulate autoimmune responses due to its high immunogenicity. The effective clearance of these NET structures in the lungs may be important to the maintenance of healthy airways. Surfactant proteins A and D are innate immune proteins in the lungs that have shown to be important in the clearance of DNA and may also be important in the clearance of NETs. SP-D is also important in minimizing the production of anti-DNA autoantibodies, which may be protective against NET-mediated autoimmunity. While SP-D can bind to NETs, its role in NET clearance and in treating NET accumulated lungs is unknown. At present, DNase is the only clinically used treatment in targeting the NET structures of these NET-filled inflammatory lung diseases. The homeostasis between NET formation and clearance is essential in sustaining a healthy immune defense against potential pathogens that are constantly in contact with our lungs. The discovery and development of compounds that can help regulate NET formation and clearance would be highly beneficial in designing therapies for these diseases.

### Conflict of interest statement

The authors declare that the research was conducted in the absence of any commercial or financial relationships that could be construed as a potential conflict of interest.
